# CD30 on extracellular vesicles from malignant Hodgkin cells supports damaging of CD30 ligand-expressing bystander cells with Brentuximab-Vedotin, *in vitro*

**DOI:** 10.18632/oncotarget.8864

**Published:** 2016-04-20

**Authors:** Hinrich P. Hansen, Ahmad Trad, Maria Dams, Paola Zigrino, Marcia Moss, Maximilian Tator, Gisela Schön, Patricia C Grenzi, Daniel Bachurski, Bruno Aquino, Horst Dürkop, Katrin S Reiners, Michael von Bergwelt-Baildon, Michael Hallek, Joachim Grötzinger, Andreas Engert, Adriana F Paes Leme, Elke Pogge von Strandmann

**Affiliations:** ^1^ Department of Internal Medicine I, University of Cologne, Cologne, Germany; ^2^ Department of Biochemistry, University Kiel, Kiel, Germany; ^3^ Department of Dermatology, University of Cologne, Cologne, Germany; ^4^ BioZyme Inc., Apex, North Carolina, USA; ^5^ Brazilian Biosciences National Laboratory, LNBio, CNPEM, Campinas, Brazil; ^6^ Pathodiagnostik Berlin, Berlin, Germany

**Keywords:** Hodgkin lymphoma, extracellular vesicle, CD30, ADAM10, Brentuximab-Vedotin

## Abstract

The goal of targeted immunotherapy in cancer is to damage both malignant and tumor-supporting cells of the microenvironment but spare unaffected tissue. The malignant cells in classical Hodgkin lymphoma (cHL) selectively express CD30. They release this receptor on extracellular vesicles (EVs) for the tumor-supporting communication with CD30 ligand (CD30L)-positive bystander cells. Here, we investigated how CD30-positive EVs influence the efficacy of the CD30 antibody drug conjugate (ADC) Brentuximab Vedotin (SGN-35). The malignant cells and the EVs expressed the active sheddase ADAM10. ADAM10 cleaved and released the CD30 ectodomain (sCD30), causing a gradual depletion of SGN-35 binding sites on EVs and creating a soluble competitor of the ADC therapy. In a 3D semi-solid tumor microenvironment model, the EVs were retained in the matrix whereas sCD30 penetrated readily into the surrounding culture medium. This resulted in a lowered ratio of EV-associated CD30 (CD30EV) to sCD30 in the surrounding medium in comparison to non-embedded cultures. A low percentage of CD30EV was also detected in the plasma of cHL patients, supporting the clinical relevance of the model. The adherence of CD30EV but not sCD30 to CD30^−^/CD30L^+^ mast cells and eosinophils allowed the indirect binding of SGN-35. Moreover, SGN-35 damaged CD30-negative cells, provided they were loaded with CD30^+^ EVs.

## INTRODUCTION

Classical Hodgkin lymphoma (cHL) is characterized by a few malignant Hodgkin and Reed-Sternberg (HRS) cells, which are supported by a heterogeneous infiltrate of proinflammatory cells. Particularly the mast cell, eosinophil and macrophage count is described as a negative prognostic marker [1–4]. The HRS cells selectively express the lymphoid activation marker CD30 (TNF receptor superfamily member 8, TNFRSF8) whereas the mast cells and eosinophils the corresponding ligand (TNFSF8, CD30L, CD153). Both are transmembrane proteins. Hodgkin cells also release extracellular vesicles (EVs) containing CD30 (CD30EV) as a membrane protein [5]. In addition, CD30 is cleaved by metalloproteinases [6]. This generates primarily two fragments, the soluble ectodomain (CD30ecto or sCD30) and the remaining cell-associated transmembrane and cytoplasmic domain (CD30endo). Virtually all commercially available CD30 antibodies detect the ectodomain. In normal and malignant cells, CD30 is cleaved by the metalloproteinases ADAM10 and ADAM17 [7, 8]. In particular ADAM10 was also detected on EVs from normal lymphoid cells and various cancer cell types (www.exocarta.org) and only recently it became clear, that the enzyme might also be functional on released EVs [9].

The Hodgkin lymphoma benefits from the CD30/CD30L crosstalk. Ligation of CD30 stimulates the NF-κB survival signaling in the HRS cells [10]. Conversely, ligation of CD30L^+^ in immune cells stimulates the release of tumor-promoting factors without degranulation of cytotoxic substances [11, 12]. EVs might replace the direct cell contact, as CD30EV is also able to stimulate CD30L^+^ cells in a CD30-dependent manner [5]. By contrast, monomeric sCD30 also binds to CD30L but it is not agonistic [13]. Both released formats also bind the therapeutic CD30 antibodies or antibody-drug conjugates (ADCs) and might influence the outcome of the immunotherapy.

The CD30 antibody drug conjugate (ADC) Brentuximab Vedotin (SGN-35, Adcetris) is highly effective and safe in the treatment of CD30^+^ lymphomas. In a pivotal phase II study with patients with relapsed and refractory cHL, the overall response rate after SGN-35 treatment was 75% with 33% complete remissions [14]. SGN-35 is an ADC, which contains the anti-mitotic drug monomethylauristatin E (MMAE), covalently linked to the chimeric CD30 antibody cAC10 [15]. After CD30-specific internalization, MMAE is cleaved from the ADC and activated to harm the affected cell. Recently, it was described that SGN-35 also damages CD30-negative bystander cells [16, 17]. The authors explained this effect by small amounts of cleaved hydrophobic MMAE, which diffuse from the cytoplasm of the CD30^+^ target cell, through the membrane into the environment. Close bystander cells might non-specifically take up this cytotoxic MMAE.

Here, we suggest another hypothesis. Our data indicate that the malignant cells release CD30 on EVs. These EVs bind to CD30L on bystander cells and present additional membrane-associated CD30 sites for the binding and toxic activity of SGN-35. We suggest that this mechanism allows dual targeting of cancer and bystander cells.

## RESULTS

### ADAM10 activity on extracellular vesicles from Hodgkin lymphoma cell lines

**Figure 1:**
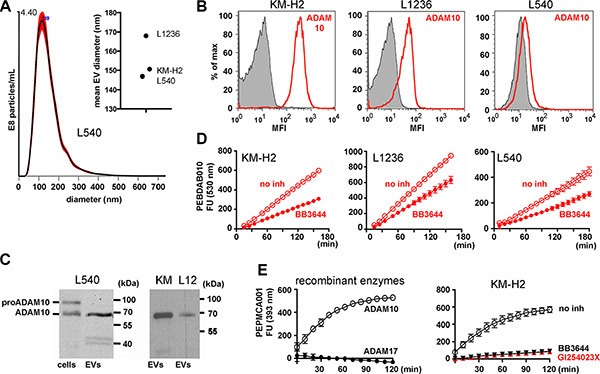
ADAM10 activity on EVs from Hodgkin lymphoma cell lines (**A**) Indicated cells (4 × 10^6^/mL) were cultivated for 2 h in serum-free medium. Under these conditions they showed no loss of viability. The cell supernatant was precleared by a sequence of centrifugation steps before it was subjected to ultracentrifugation for 2 h at 100000 × g. The pellet was suspended in 1 ml of PBS and tested by nanoparticle tracking analysis (NTA). The graph shows an overlay of 5 independent determinations of the L540 EVs. The mean diameters of EVs from all tested cell lines are shown in the summarizing graph. (**B**) The EVs from 8 × 10^7^ KM-H2, L1236 or L540 cells were immobilized at 4.5 μm-microspheres. Aliquots of the microspheres were incubated with ADAM10 antibody (red line) or isotype control (filled histogram). The beads were labeled with fluorescence-labeled anti-mouse IgG and evaluated by flow cytometry. (**C**) ADAM10 was determined by Western blotting in Triton X-100 lysate from 1 × 10^5^ L540 cells (cells) or EVs from 8 × 10^7^ L540, KM-H2 (KM) or L1236 (L12). (**D** and **E**) Aliquots of EVs from 4 × 10^7^ cells or 200 ng of recombinant ADAM10 or ADAM17 were suspended in 25 mM Tris-HCl, pH 8 containing 6 × 10^−4^ % Brij-35 in the presence or absence of 3 μM BB3644 or 3 μM GI254023X. Then, the aliquots were incubated with the fluorescent substrates PEPDAB010 (D) or PEPMCA001 (E) (BioZyme Inc., Apex, NC) in black microtiter plates at 37°C. Fluorescence was determined in a kinetic study at 530 nm (D) or 393 nm (E) as indicated. The data show means of two independent experiments minus background fluorescence without EVs.

The size of EVs from the supernatants of three cHL cell lines was determined by nanoparticle tracking analysis (NTA). The EVs from L540, KM-H2 and L1236 cells appeared with a typical mean EV diameter between 147 nm and 170 nm (Figure 1A). They expose CD30 [5]. In semi-quantitative mass spectrometry we identified the CD30 sheddase ADAM10 with one, three and four unique peptides in EVs from KM-H2, L1236 and L540 cells, respectively (Supplementary 1). ADAM10 was also detected by flow cytometry in EVs from all tested cell lines (Figure 1B). Freshly synthesized ADAM10 is functionally inhibited through its inhibitory propeptide (aa 1-223, Supplementary 1). It is activated by the proteolytic removal of the propeptide. In mass spectrometry of EVs we only identified unique hits from mature ADAM10 (Supplementary 1). In cell lysate, we identified both, pro-ADAM10 and mature ADAM10 by Western Blot (Figure 1C). In EVs we confirmed only the mature form. These data suggest that active ADAM10 is expressed on EVs.

To directly demonstrate that ADAM10 is active on EVs, we applied fluorescence resonance energy transfer metalloproteinase substrates to isolated EVs. A peptide, based on the precursor of TNF-alpha (PEPDAB010), was efficiently hydrolyzed by the EVs from KM-H2, L1236 and L540 (Figure 1D). The metalloproteinase inhibitor BB3644 blocked the cleavage, confirming the metalloproteinase activity on EVs. Since PEPDAB010 is also sensitive for other ADAM enzymes, we additionally tested the ADAM10-selective PEPMCA001. The latter is very selective for ADAM10 over other enzymes and it was not cleaved by recombinant ADAM17 (Figure 1E) [18]. EVs of KM-H2 cells efficiently processed this ADAM10 substrate and both BB3466 and the ADAM10-selective inhibitor GI254023X blocked the cleavage (Figure 1E) [19]. Based on these data, we conclude that catalytically active ADAM10 is expressed on EVs of cHL cell lines.

### CD30 ectodomain shedding on isolated EVs

To study CD30 ectodomain shedding on isolated EVs, we used a pair of non-competing CD30 ectodomain (CD30ecto) antibodies (Ki-2 mAb and Ki-3 mAb; used in commercial ELISA) and generated a novel pair of CD30 endodomain (CD30endo) antibodies (Ki-10 mAb and Ki-12 mAb; Supplementary 2). The novel Ki-12 mAb binds to the cytoplasmic sequence aa 465-500 and the novel Ki-10 mAb to the far C-terminal sequence of CD30 (aa 500–595). In addition to the published bands of intact CD30 [20], the CD30endo antibodies detected also smaller bands in Western Blot. They might at least in part represent degradation products of CD30, which are expected after CD30 cleavage. In cHL cell lines CD30endo is biosynthesized as the C-terminal part of CD30. However, a splice variant of CD30 (variant CD30; aa 464–595) has been described in other cell types [21, 22]. Therefore the epitopes of the novel antibodies might not be cHL-selective. However, similar to the established CD30ecto antibody Ki-2, the CD30endo antibodies showed strong staining of the malignant cells in cHL tissue. Because of the so far unknown selectivity, we used the novel antibodies only to support ectodomain shedding on EVs.

**Figure 2:**
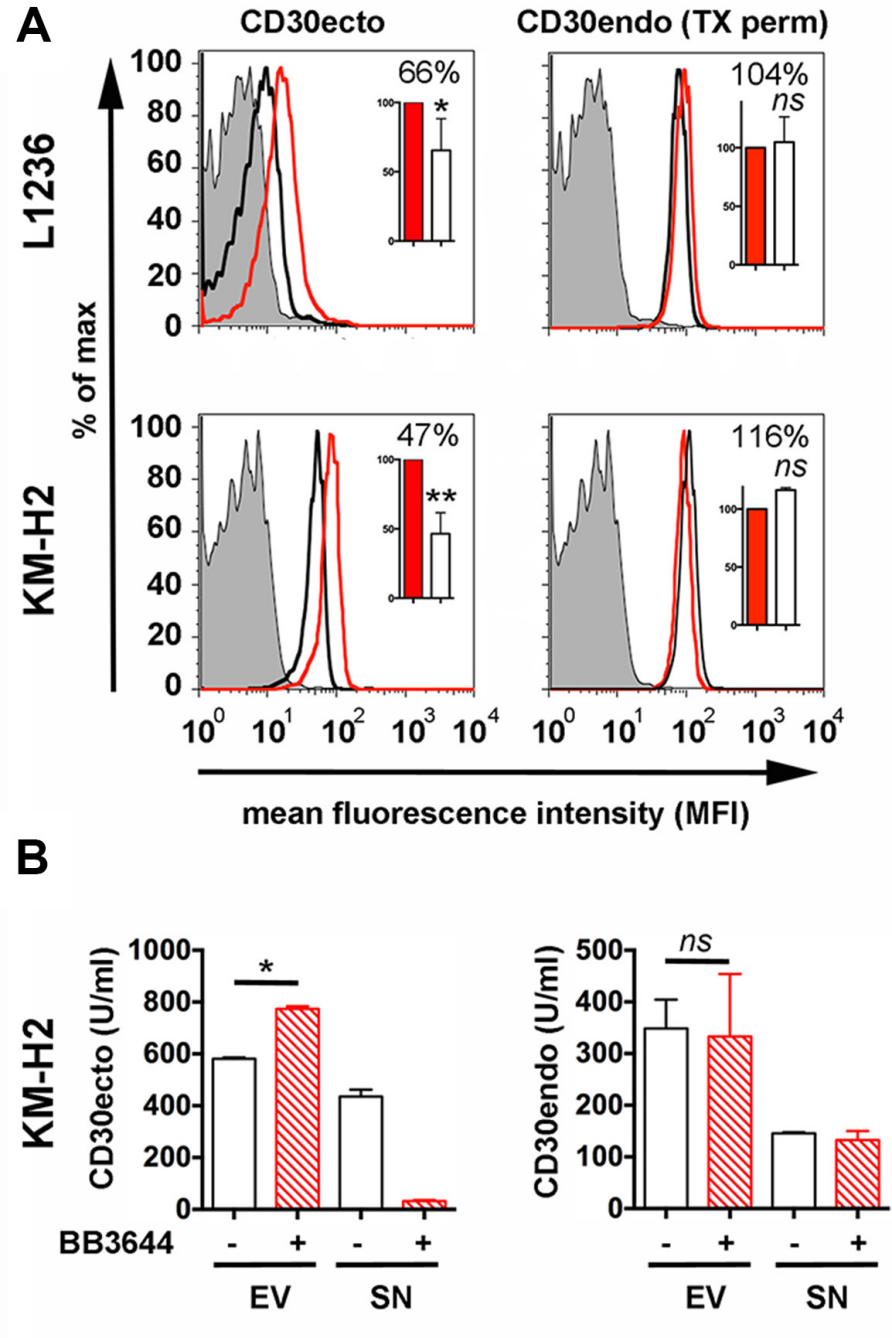
Ectodomain shedding on Hodgkin cell-derived EVs Isolated EVs from the supernatants of 8 × 10^7^ L1236 and KM-H2 cells were cultivated in EV-depleted medium for 18 h at 37°C ± BB3644 (3 μM, red line and red/shaded bar). Then, EVs were again centrifuged at 100000 × g. (**A**) The pellet was washed once in PBS and immobilized at 4.5 μm-microspheres. Aliquots of microspheres were incubated with Ki-2 mAb (CD30ecto), Ki-12 mAb (CD30endo) or isotype control (filled histogram). Aliquots for CD30endo determination were permeabilized with 0.1% Triton X-100 before the addition of the antibodies (TX perm). Samples were labeled with fluorescence-labeled anti-mouse IgG for flow cytometry. (**B**) After ultracentrifugation, the pellets and the supernatants were evaluated by the commercial ELISA (CD30ecto) and the CD30endo ELISA as indicated. The mean fluorescence intensity (MFIs) of 4–5 independent experiments were evaluated by arbitrarily setting the inhibited aliquots as 100%. The percentages of the non-inhibited aliquots were statistically evaluated by a two-tailed, nonparametric *t*-test (Mann-Whitney) (ns = not significant, * = *P* < 0.05, **P < 0.01).

We incubated EVs from KM-H2 and L1236 cells for 18 h and determined CD30 by flow cytometry. In this period EVs showed a significant reduction of CD30ecto to 47% and 66% of the inhibited control but no reduction of CD30endo (Figure 2A). CD30endo remains at the cytoplasmic side of the membrane after cleavage. The uninfluenced CD30endo confirms that the CD30ecto loss is due to ectodomain cleavage. We also calculated the ratio between the extracellular and intracellular CD30 levels (Ki-2Ab/Ki-10Ab) from the geomeans of flow cytometry. L1236 EVs resulted in ratios of 0.084 versus 0.163 and KM-H2 in 0.423 versus 0.862, in untreated and inhibited aliquots, respectively. This equals a CD30endo-normalized CD30ecto reduction to 51.5% (L1236 EVs) and 49.1% (KM-H2 EVs).

We developed a CD30endo ELISA using the novel antibodies Ki-10 and Ki-12. Together with the commercial ELISA (CD30ecto) we were able to detect and quantify the intracellular and extracellular part of CD30 (Figure 2B). ELISA data confirmed that isolated EVs released the CD30ecto (sCD30) into the supernatant. This depleted the EV-associated CD30ecto signal but kept the amount of CD30endo stable. We also calculated the ratio of extracellular and intracellular CD30 units/mL. With a ratio of 1.675 for untreated and 2.35 for inhibited EVs, we calculated a CD30endo-based CD30ecto loss to 71.3% compared with the metalloproteinase-inhibited control. A background of CD30endo was detected in the supernatants after ultracentrifugation at the end of the incubation time. This might at least in part be explained by the incomplete EV sedimentation under 2 h ultracentrifugation. Repeated centrifugation, longer centrifugation or higher gravidity better depletes EVs but the EV decomposition is also enhanced [23]. However, both tests clearly indicate that CD30 is also shed on EVs and that the CD30 reduction is caused by ectodomain cleavage by metalloproteinases.

### Release of CD30 in matrigel microenvironment model

In cHL, the HRS cells are surrounded by bystander cells and a non-cellular matrix. Nodular sclerosis (NS) is the most common cHL subtype (~80%) and displays a strong extracellular matrix (ECM) deposition [10, 24]. Thus, the EVs have to overcome a microenvironment of ECM and bystander cells to reach the circulation. This raises the question whether EVs loose the CD30 ectodomain by metalloproteinase cleavage during migration through this microenvironment. We first tested the influence of semi-solid matrigel, which contains proteins of the ECM and the basal membrane but does not respect binding of EVs to bystander cells. In a second approach, we investigated the influence of cell aggregates (Supplementary 3).

**Figure 3:**
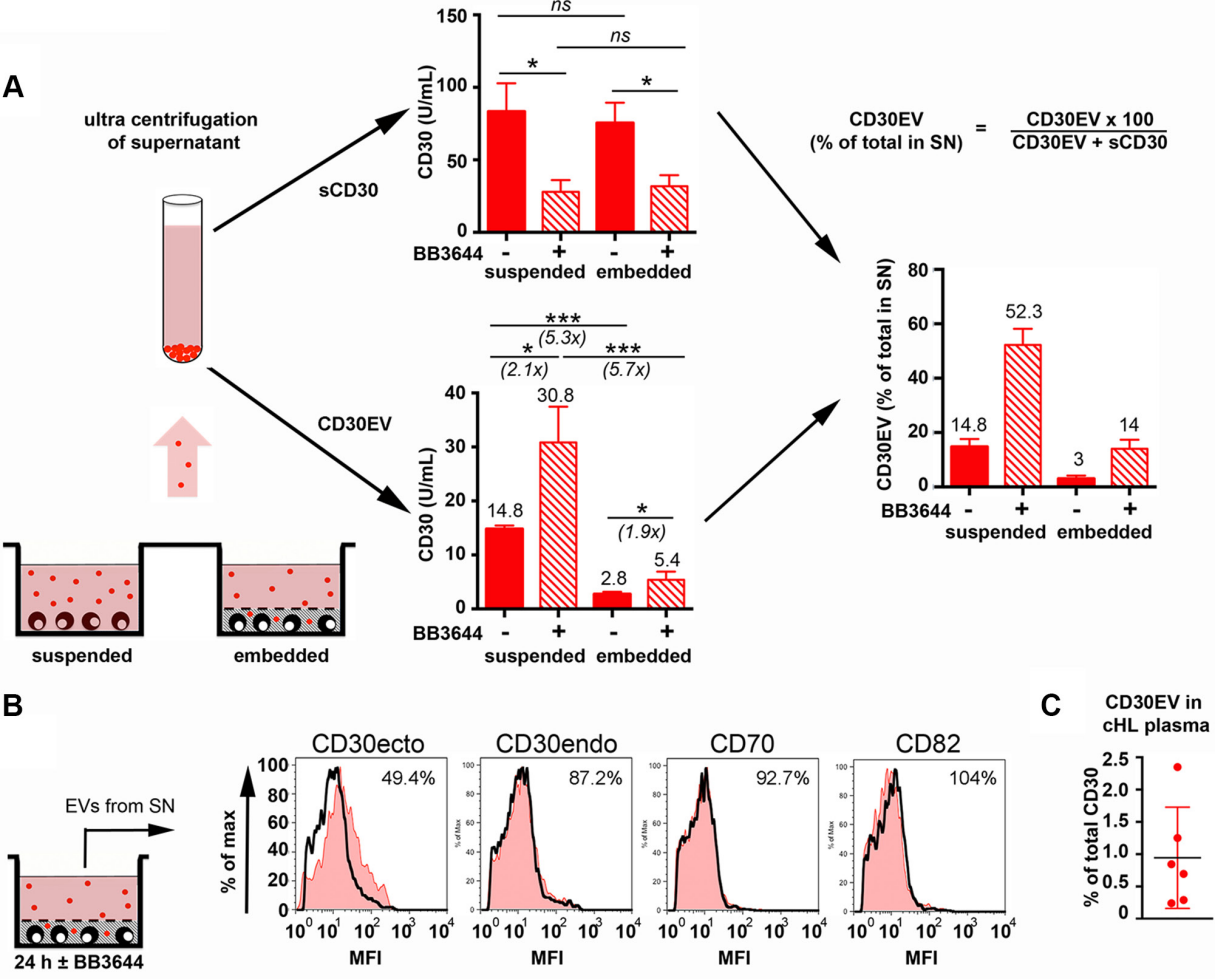
Release of CD30 in 3D microenvironment model (**A**, **B**) L540 cells (2 × 10^5^) were embedded in a 24-well plate in 100 μL of a semi-solid gel containing equal volumes of growth factor-reduced matrigel and RPMI-1640 with 20% EV-depleted FCS ± BB3644 (3 μM) (embedded). After hardening of the matrix, 900 μL of culture medium containing 10% of EV-depleted FCS (± 3 μM BB3644) was added. As control, cells were cultivated in suspension on top of 100 μL of cell-free semi-solid matrigel (suspended). After 24 h, the culture supernatants, without matrigel, were removed and pre-centrifugated to remove cells and cell debris. Then, the supernatant was ultracentrifugated at 100000 × g for 120 min to sediment the EVs (•). The ultracentrifugation supernatant was isolated and the EV pellet was suspended in PBS and adjusted to the same volume as the supernatant. (A) The CD30 ectodomain ELISA was used to determine CD30EV in the PBS-suspended EVs and sCD30 in the ultracentrifugation supernatant. The results show U/mL as means ± SE for four independent experiments. From these experiments the percentage of CD30EV was calculated (% of total released CD30). (B) EVs were immobilized at 4.5 μm-microspheres and aliquots of microspheres were incubated with antibodies as indicated and investigated by flow cytometry. The mean fluorescence intensity (MFI, black graph) was determined and compared with the BB3644-inhibited aliquots (red tinted histograms). The inhibited samples were arbitrarily set as 100%. (**C**) The percentage of CD30EV (% of total released CD30) was determined the plasma of cHL patients (*N* = 6).

We embedded L540 cell (NS-subtype) and used CD30 as a tracer to study the EV migration and CD30 shedding during the passage through matrigel (Figure 3A). CD30EV and sCD30 were separated by ultracentrifugation. Then, we compared their amounts in the medium of a suspension cell culture and in the medium that surrounds the matrigel-embedded culture. Embedding did not significantly influence the release of sCD30 in the surrounding supernatant indicating that CD30 cleavage and sCD30 diffusion was not considerably inhibited in the matrix. In contrast, embedding resulted in a 5.3-fold decrease of released CD30EV. This equals a reduction to 19% of the suspended control (*P* > 0.0001, *N* = 4) and a drop in the percentage of CD30EV from 14.8% in the supernatant of suspended cells to 3.0% in embedded cells. This reduction of CD30EV in the supernatant of embedded cultures might be due to a general EV retention in the matrix and under retention, EVs might shed CD30 like the suspended EVs. Only comparing metalloproteinase inhibited aliquots, we measured 5.7-fold more CD30EV (*P* = 0.0003, *N* = 4) in the supernatants of suspended than embedded aliquots, clearly indicating that EVs are strongly retained in the matrix. However, when we evaluated the effect of metalloproteinases on matrigel embedded aliquots, we measured 1.9-fold more CD30EV (*P* = 0.0153, *N* = 4) under inhibition. This indicates that EVs loose CD30ecto by ectodomain shedding under the observed period of time. Thus, strongly CD30ecto-depleted EVs leave the matrix. This depletion was also true for the ADAM10 substrate CD44 (not shown) but not for shedding-insensitive substrates. As shown by flow cytometry, CD30 lost approximately 50% of its ectodomain. In contrast, the shedding remnant cytoplasmic portion of CD30 (CD30endo) or the metalloproteinase-resistant tetraspanin CD82 and the TNF superfamily member CD70 (TNFSF7, CD27L) remained stable on EVs (Figure 3B). Thus, the EVs suffer a loss of ADAM10-sensitive ectodomains under migration through the matrigel matrix.

Similar data were received in another model with CD30^+^ cells, this time disseminated in large cell aggregates. This model rather reflects the retention of EVs by bystander cells. The aggregates released a higher amount of EVs and a higher percentage of CD30EV in the surrounding medium when they were suspended before EV isolation. These data indicate that CD30^+^ EVs from large aggregates of normal cells are also retained by surrounding bystander cells and perform ectodomain shedding (Supplementary 3).

To test whether our models reflect the release of CD30 in cHL tissue, we also determined the percentage of CD30EV in the plasma of cHL patients from the HD16 study. These patients did not receive antibody treatment. Here we found an even lower percentage of CD30EV (0.9%, range: 0.2–2.3%, *N* = 6) than in the supernatant of embedded cultures (Figure 3C). CD30, is a selective marker of cancer cells in cHL tissue and the CD30 serum levels correlate with the tumor load. We therefore speculate that sCD30 and CD30EV in the serum originates predominantly from the tumor. Because of the low percentage of CD30EV in the serum, we take it as indirect evidence that most CD30 is degraded from EVs before reaching the circulation.

### CD30EV guides SGN-35 to cells of the microenvironment

**Figure 4:**
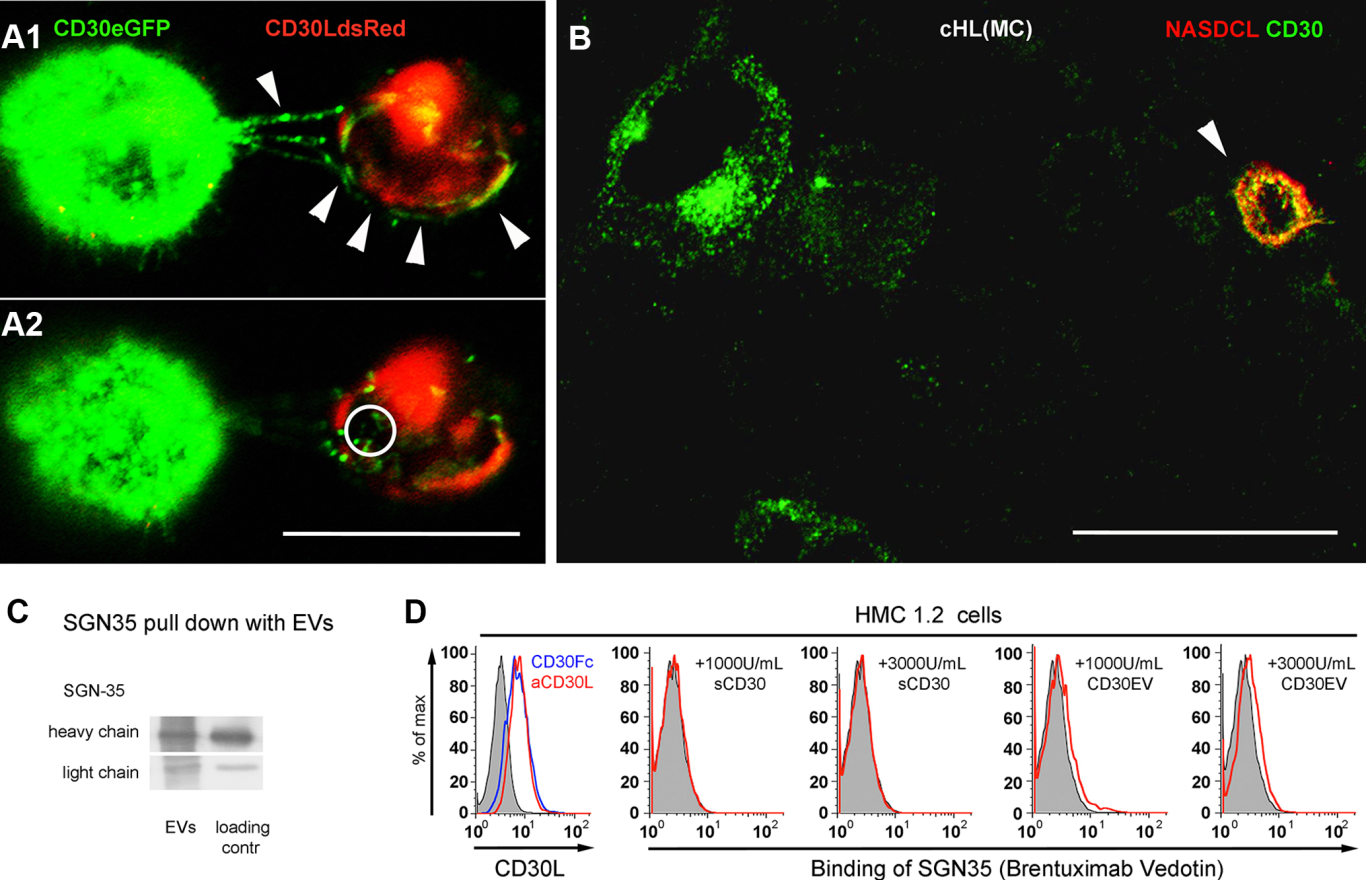
CD30-positive EVs target SGN-35 to cells of the microenvironment (**A**) CD30L-DsRed2-transfected HMC 1.1 (red) and CD30-eGFP-transfected HD-MyZ (green) were co-cultured in growth factor-reduced matrigel and incubated for 2 h at 37°C, 5% CO2. Two consecutive confocal images (Δ = 1.219 μm) of co-cultivated cells are shown. Arrowheads indicate release and binding of CD30^+^ vesicles to CD30L^+^ HMC 1.1 cells. The circle indicates internalized CD30. (**B**) Confocal image of a tissue section of a lymph node infiltrated by cHL of mixed cellular subtype was stained with NASDCL (red) and with a CD30 primary antibody (Ber-H2) and an ALEXA488-conjugated secondary antibody (green). Bars indicate 30 μm. Confocal images were taken with laser scanning microscopy (Zeiss Meta 510, Zeiss, Germany) using a 40× oil objective with NA 1.3 and the appropriate filters and analyzed with Imaris 7.0 software. (**C**) L540 cells (5 ml of 2 × 10^6^/mL) were cultivated for 3 h at 37°C in serum-free medium with biotin-labeled SGN-35 (1 μg/mL). Supernatants were harvested, precentrifuged and EVs were finally pelleted at 100000 × g. The pull-down of SGN-35 on pelleted EVs was investigated by Western Blot under reducing conditions. Streptavidin-coupled peroxidase was used to detect the heavy and light chain of the biotinylated SGN-35 (EVs). Biotinylated SGN-35, directly applied to the Western Blot served as loading control. (**D**) Determination of CD30L on HMC1.2 cells by flow cytometry. HMC1.2 cells (5 × 10^5^/mL) were incubated for 1 h on ice with CD30Fc or an anti-CD30L antibody (left) or with the indicated amounts of sCD30, CD30EV or without CD30 (tinted curve) in the presence of 0.1 μg/mL of FITC-labeled SGN-35.

Next we tested whether cancer cell EVs transport CD30 to adjacent immune cells and as a consequence allow SGN-35 binding to CD30-negative cells of the neighborhood. In a 3D matrigel co-culture of CD30^+^ (green) and CD30L^+^ cells (red), vesicle-associated CD30 (CD30EV) was released from the donor cell. Confocal images demonstrate that the EVs bind to the surface of the CD30L^+^ cell (Figure 4A1; white arrow heads) and a minor portion of the CD30^+^ EVs is taken up (Figure 4A2; white ring). We also tested the migration of CD30 to tumor-supporting bystander cells in cHL tissue. Therefore naphthol AS-D chloroacetate esterase (NASDCL, red) and CD30 (ALEXA 488, green)-positive cells were co-stained in a 30 μm section of a cHL-affected lymph node of mixed cellularity sub-type (MC). NASDCL stains predominantly neutrophils, which express CD30L but not CD30 [12, 25]. The confocal image shows a strong co-localization of CD30 to a NASDCL^+^ bystander cell (arrow head), indicating that CD30 is migrating to bystander cells *in vivo* as well (Figure 4B).

Next, we tested if CD30^+^ cells release EVs that bind and transport SGN-35. Therefore the CD30^+^ cHL cell line L540 was cultivated with biotin-labeled SGN-35, washed and further cultivated in serum-free suspension culture. The EV fraction from the supernatant was isolated and washed by ultracentrifugation. Then, we tested the purified EVs for the presence of SGN-35. As shown in Figure 4C, these EVs contained both the biotinylated heavy and the light chain of SGN-35 indicating that EVs from cancer cells bind and transport SGN-35 (Figure 4C).

The mast cell line HMC1.2 is CD30-negative but expresses the CD30 ligand and binds CD30 (Figure 4D, left). 458 ± 190 U/mL of CD30 were detected in the serum of cHL patients with progressive disease or relapse [26]. We expect higher concentrations in the malignant tissue, at the site of origin. Therefore we tested SGN-35 binding along with sCD30 or CD30EV, both adjusted to 1000 or 3000 U/mL. SGN-35 alone did not bind to the cells but SGN-35 was binding together with CD30EV. sCD30 was unable to link SGN-35 to HMC-1.2 cells. These data suggest that cancer cell-derived CD30EVs but not monomeric sCD30 enable the binding of the anti-CD30 ADC to CD30^−^ but CD30L^+^ cells, *in vitro.* Together, these data support the hypothesis that CD30^+^ EVs from cancer cells allow the off-target binding of SGN-35 to bystander cells of the tumor microenvironment.

### SGN-35 damaged CD30L^+^ immune cells through CD30EV

**Figure 5:**
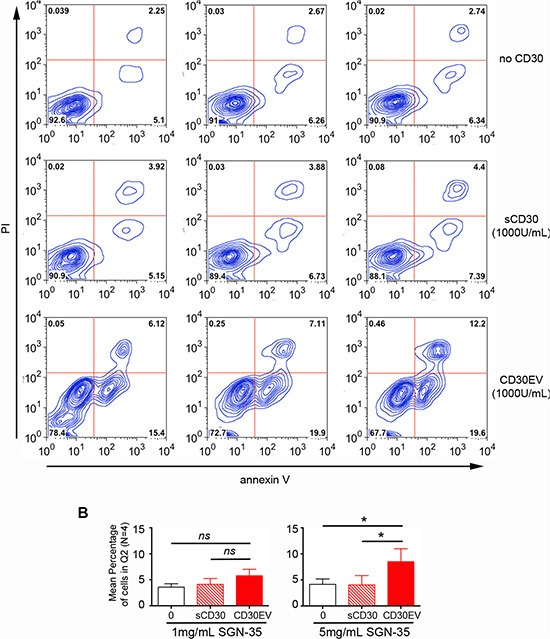
SGN-35 damaged CD30L^+^ immune cells through CD30EV EOL-1 cells (5 × 10^5^/mL) were cultivated for 96 h with SGN-35 (0, 1 or 5 μg/mL) and ± 1000 U/mL sCD30 or 1000 U/mL CD30 on EVs (CD30EV) from L540 cells. Cells were stained with annexin V-coupled ALEXA647 and propidium iodide (PI). They were analyzed by flow cytometry and double-positive cells were gated (Q2). The percentage of cells/gate is indicated. (**A**) The image shows one representive experiment of four. (**B**) The mean percentage in Q2 ± SD of four independent experiments is depicted for some conditions as indicated. The significance was determined by two-tailed, non-parametric, Mann-Whitney *U* test (* = *P* < 0.05, > 0.01).

Next, we studied whether SGN-35 is able to damage CD30^−^/CD30L^+^ EOL-1 cells with the help of CD30EV. As indicated by an increase of the propidium iodide (PI) staining, CD30EV increased the percentage of cells in Q2 in comparison to aliquots without CD30 (Figure 5A and 5B). Thus, CD30EVs raised the mean %-age ± SD from 3.57 ± 0.66 to 5.79 ± 1.23 for 1 μg/mL of SGN-35 and from 4.15 ± 1.03 to 8.51 ± 2.46 for 5 μg/mL (*N* = 4). Although the %-age clearly increased also with 1 μg/mL SGN-35, only the experiments with 5 μg/mL SGN-35 were statistically significant (Figure 5B). In contrast, co-incubation with sCD30 was almost ineffective in this respect. Apoptosis is measured by the expression of phosphatidylserine (PS) on the outer leaflet of the target cell. EVs generally show a constitutive expression of PS. This explains the elevated background with annexin V staining, when EVs were applied.

## DISCUSSION

### Ectodomain shedding on EVs

We are only beginning to understand the whole range of EV functions. In cancer, opposing effects such as suppression of malignant cells or stimulation of metastasis have been described [27, 28]. Depending on the type and activation status of the releasing cell, variable amounts of EVs are liberated with a variable load of proteins and nucleic acids. Whereas luminal microRNA influences the fate of the recipient cell, the surface proteins are responsible for the contact with the environment. As an example, the integrin pattern on the surface of EVs participates in the organotropism in metastasis [29]. Many adhesion proteins and membrane-anchored receptors are cleaved by ADAM proteinases [30, 31]. Other membrane proteins are quite resistant such as CD70, CD82 or tetraspanins and often serve as marker to identify EVs [32]. Here, we demonstrated catalytically active ADAM10 and the cleavage of CD30 on isolate EVs. Whereas cells are able to substitute shed membrane proteins, EVs cannot synthesize and replace the cleaved protein. This leads to a slow-acting depletion of metalloproteinase-sensitive proteins.

**Figure 6:**
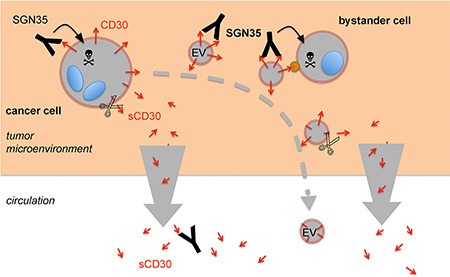
Proposed model for the role of EVs and CD30 shedding for the immunotargeting with SGN-35 The malignant cells in cHL reside in lymphoid tissue, surrounded by a microenvironment of extracellular matrix and proinflammatory cells. They selectively express the receptor CD30. The CD30 antibody-drug conjugate SGN-35 binds to the CD30^+^ tumor cells, is internalized and the toxic compound is cleaved and activated by lysosomal proteases. The malignant cells not only expose CD30 on the surface, they also release CD30 on EVs (CD30EV), either by membrane blebbing or release of exosomes from multivesicular endosomes. EVs also bind SGN-35 and SGN-35-loaded EVs migrate away from the cancer cell. The loading of EVs might also occur within the tumor cell. After SGN-35 internalization, the drug might target to multivesicular endosomes instead of lysosomes. Apoptotic blebs of damaged tumor cells might contribute to the release of SGN-35-loaded EVs. Mast cells and eosinophils support the cHL tumor growth. These cells express the natural CD30 ligand and bind SGN-35-loaded CD30EV. These cells might internalize the EVs and are damaged by SGN-35 in a CD30EV-dependend manner. Cells and EVs express the CD30 sheddase ADAM10, which gradually cleaves the ectodomain. In the microenvironment, sCD30 is quickly drained whereas EVs are retained. Monomeric sCD30 is a competitor of SGN35 binding to cells and EVs. This hypothesis suggests an elevated ratio of membrane-associated CD30 within the matrix and an elevated ratio of sCD30 outside of the matrix. SGN-35 exploits this mechanism to preferentially target cancer and bystander cells in the tumor microenvironment. The CD30-depleted EVs and the high level of competing sCD30 in the circulation might explain the minute side-effects of the drug.

In tissue, when EVs diffuse from the releasing cell, this time-dependent loss of cleavable proteins might also result in a loss-of-function gradient. In contrast, the functions of shedding-resistant proteins persist even distant from the releasing cell (Figure 6). This hypothesis has consequences for a use of EVs as biomarker in body fluids. Instable ectodomains of proteins are unreliable biomarkers in body fluids. Instead, stable tumor-characteristic proteins and in particular the luminal microRNA are more reliable to monitor the progression of a distant malignancy. Thus, characteristic RNA and the tumor-specific EGFRvIII could be detected in serum EVs of glioblastoma patients [33]. The prognostic value of such serum EVs is currently under investigation [34]. Although EVs were first described in cHL, there is to the best of my knowledge no study available on the prognostic value of serum EVs in cHL [35]. Recently, circulating cell-free microRNA was suggested to have a prognostic value in cHL but association with EVs was not shown [36]. CD30endo might be a promising plasma biomarker, because it seems to be stably detectable by both novel antibodies, even after ectodomain cleavage. However, CD30endo is problematic for another reason. A splice variant of CD30, comprising only the CD30 cytoplasmic domain is not a typical cHL protein. It is found in other cells, such as alveolar macrophages, acute myeloid leukemia (AML), myeloproliferative disorders (MBC) and chronic lymphocytic leukemia (CLL) [21]. We are currently investigating CD30endo as a plasma EV biomarker for cHL and other diseases.

### Role of CD30EV in bystander cell targeting by SGN-35

The desired function of SGN-35 is to bind to CD30 on cancer cells and subsequently kill them. CD30EV and sCD30 bind SGN-35 and compete with tumor cell CD30. The competition impact was not tested and can only be estimated. The released antigens are probably different in the strength of their antibody binding. sCD30 is monomeric and exposes only one epitope. The simultaneous binding of two linked epitopes such as cell or EV-associated CD30 results in much stronger binding forces. Therefore, SGN-35 might favor antigens that are membrane-associated. Consequently, CD30EV is a stronger competitor than sCD30. It appears from the confocal images that CD30EV concentrates around the CD30L-positive bystander cells. These sites are expected to bind SGN-35 with a similar avidity as the CD30^+^ cancer cells. Cells with strong CD30 expression yield 1000 U CD30 from 10^6^ cells [6]. We were only able to isolate 70 U CD30 on EVs (14 U from 2 × 10^5^ cells). Therefore, CD30EV is a probably a competitor with high avidity but there is less CD30EV than cell-associated CD30 in tissue. However, it is not only the avidity of the antigen that counts. High concentrations of sCD30 might reduce the SGN-35 binding to the major antigens. In SCID mice with a L540 xenotransplant, metalloproteinase inhibition reduced the serum sCD30 level significantly and improved the tumor therapy with a CD30 immunotoxin [37].

All CD30 antibodies raised against the ectodomain define three major serological clusters (A-C) [38]. The murine antibody backbone of SGN-35 (C10) was allocated to cluster C, along with the inhibitory (HeFi-1) and the agonistic (M44) CD30 antibodies [38], suggesting that the SGN-35 epitope is involved in the ligand binding or close to it. In contrast to Ki-1 mAb (cluster B), which binds to an epitope distant from the CD30L binding site, SGN-35 was not able to bind to CD30L by sCD30 bridging (Figure 4) [7]. Thus, the ADC can only bind to CD30L^+^ cells, when they provide free SGN-35 epitopes, as EVs can do. This might explain the failure of sCD30 to communicate SGN-35 binding to bystander cells.

To the best of my knowledge, this is the first study that presents the hypothesis that EVs help to damage bystander cells by translocation of a tumor-associated antigen. So far, we focused on the CD30/CD30L axis and on cHL, because CD30 is very selective for certain diseases and CD30L is expressed on cHL-typical tumor-supporting bystander cells. However, EVs do not transport CD30 alone. Mass spectrometry of EVs from cHL cell lines identified various integrins, adhesion protein (not shown). Such molecules might participate as co-receptors in EV binding because the integrin pattern also determines the organotropism of EVs in glioblastoma [29].

The CD30/CD30L axis is not selective for cHL. In adults, CD30 is also overexpressed in other malignancies, such as anaplastic large cell lymphoma (ALCL), cutaneous T-cell lymphoma (Sézary syndrome, Mycosis fungoides), diffuse large B-cell lymphoma (DLBCL) and certain autoimmune diseases. Some of which are evaluated in clinical studies with SGN-35 [39]. Because most cells release EVs it is possible that CD30EV is also released in these cases, but it is not shown yet. The presence of tumor-supporting CD30L-positive bystander cells is another question. In addition to mast cells and granulocytes, CD30L is also expressed on subsets of activated T and B cells [12, 40, 41]. The EV interaction with the latter was not tested so far. Nevertheless, a mast cell infiltrate has been described in DLBCL [42] and hypereosinophilia are shown in different T-cell lymphomas [43]. The treatment of such malignancies might profit from the EV-based cross-fire effect of SGN-35.

Together, we provide a hypothesis that explains the powerful clinical efficacy of SGN-35 (Figure 6). The anti-CD30 ADC SGN-35 is not only able to bind and damage CD30^+^ cancer cells but also CD30^−^ cells when loaded with CD30^+^ EVs from the cancer cell. However, EVs gradually loose this mobile targeting structure by ectodomain shedding on the pathway through the matrix or cell aggregates to the circulation. We suggest that this loss confines the bridging functionality of EVs to cells of the close microenvironment of the cancer cells. This model explains the high anti-tumor and low systemic efficacy of SGN-35.

## MATERIALS AND METHODS

### Cells and reagents

The CD30^+^ cHL cell lines L540, L428, KM-H2 and L1236, the CD30^−^ cHL cell line HD-MyZ and the acute myeloid (eosinophil) leukemia line EOL-1 were purchased from DSMZ (Braunschweig, Germany). The mast cell line HMC-1 was from Dr. J. Butterfield (Mayo Clinic, Rochester MN). Cells were cultivated in RPMI-1640 or IMDM (HMC-1) containing 10% FCS, penicillin, streptomycin and 50 μM β-mercaptoethanol. EV-depleted FCS was generated by overnight centrifugation at 100000 × g. The CD30 monoclonal antibodies Ki-2, Ki-3 were generated as described [38, 44, 45]. The goat anti-human Fc Ab (GaH-IgG) was from Dianova (Hamburg, Germany) and the metalloproteinase inhibitor (BB3644) was from British Biotech Pharmaceuticals Ltd. (Oxford, U.K.). The ADAM10 inhibitor GI254023X was kindly provided by Dr. A Ludwig, Aachen, Germany. The fluorescence resonance energy transfer metalloproteinase substrates PEPDAB010 and PEPMCA001 were from BioZyme Inc. (Apex, NC). Peripheral blood mononuclear cells were isolated from buffy coats from the local blood bank. The testing of plasma samples from cHL patients was approved by the local Ethics Committee (HD16 study, reference 09/039).

### Determination of CD30endo by ELISA

Microtiter plates (MaxiSorb, Nunc) were coated with 50 μL of Ki-12 mAb (10 μg/mL in sodium carbonate buffer, 50 mM, pH 9.2) by overnight incubation at 4°C. The plates were washed with PBS and blocked with PBS containing 5% milk powder for 1 h and subsequently stored at −20°C until used. After triplicate washing with PBS containing 0.02% Tween 20 (PBS/Tween 20), serial dilutions of a CD30 standard and the CD30-containing samples were added and incubated at RT for 2 h. Plates were washed four times with PBS/Tween 20 and 50 μL of biotin-labeled Ki-10 mAb was added and incubated for 1 h at RT. After washing, the plates were incubated for 40 min with 50 μL of streptavidin-horseradish peroxidase conjugate, washed and peroxidase activity was developed with TMB solution (Thermo Fisher). The reaction was stopped with 0.16 M sulfuric acid. The absorbance was measured at 450 nm.

### Plasmids

Construction of the CD30L-DsRed2 plasmid: Human CD30L (CD153, isoform 1) was amplified by PCR from the cDNA of DG75 cells using the *Bg*lII forward primer *5*′-actcagatctcgagGAATGGACCCAGGGCTGCAGCAA GCA-*3*′ and the *Bam*HI reverse complement primer *5*′-tgagaggatccTCAGTCTGAATTACTGTATAAGAAGA TGGACA-*3*′ *to* clone the PCR product into the *Bgl*II and *Bam*H1 restricted expression vector pDsRed2-C1 (Clontech, CA). Construction of the CD30-eGFP expression plasmid: The CD30 cDNA was amplified from CD30-pcDNA3 plasmid [46] using the *Hind*III forward primer 5′-gcgagaagcttATGCGCGTCCTCCTCGCCGCG-3′ and the *Bam*HI reverse complement primer 5′-tcgtaggatccCCCTTT CCAGAGGCAGCTGTGGG-3′. This PCR product was cloned into the *Hind*III/*Bam*HI restricted plasmid pEGFP-C1 (Clontech CA).

### Transfection

HD-MyZ cells (1 × 10^5^ /ml) were allowed to adhere; HMC-1.1 cells were immobilized and cultivated on a fibronectin-coated surface (15 μg/ml). Medium was removed and 400 μl medium (RPMI-1640 or IMDM medium, respectively) + 2% FCS was added. DNA (0.8 μg DNA/50 μl Opti-MEM I; Invitrogen) and Lipofectamine 2000 (1 μl/50 μl Opti-MEM I; Invitrogen) were prepared separately and incubated for 5 min. Then, DNA and Lipofectamine solutions were mixed and incubated for 20 min before being added to the cells. Cells were incubated for 24 h before use. After 12 h, the medium was exchanged and cells were cultivated in their standard medium. This protocol was up-scaled to transfect more cells.

### Vesicle isolation

Cells were washed and cultivated under serum-free conditions at 4 × 10^6^/mL for 2 h. Cell supernatants were collected and cleared by five consecutive centrifugation steps, i.e. 5 min at 200 × g, 15 min at 2000 × g, 2 times 15 min at 3500 × g and finally 30 min at 10000 × g. Cleared supernatants were sedimented in an ultracentrifuge, for 2 h at 100000 × g. Pellets and the final supernatants were collected. Vesicle fraction-containing pellets were washed with PBS by ultracentrifugation for two hours.

### Flow cytometry

HMC 1.2 or EoL-1 cells (5 × 10^5^/ml) were incubated for 1 h on ice with sCD30, ultracentrifugation-enriched CD30-containing EVs or HBSS buffer alone. After washing with PBS containing 1% albumin and 0.1% sodium azide, cells were incubated for another 30 min on ice with FITC-coupled SGN-35 (0.1 μg/ml). After washing in the above PBS, cells were evaluated by flow cytometry. Vesicles alone were incubated overnight with polybead carboxylate microspheres (4.5 μm; Polysciences INC, Warrington, PA). The beads were blocked with 1% BSA (v/w) in PBS. Then, aliquots were incubated with unlabeled or fluorescence-labeled antibodies. Aliquots with unlabeled antibody were in a second step labeled with fluorescence-labeled anti-mouse IgG (Dianova, Hamburg, Germany). Beads were evaluated by flow cytometry.

### Toxicity of SGN-35

Cells were incubated for 96 h with sCD30, CD30EV and SGN-35 as indicated. Then, cells were washed and incubated for 15 min at room temperature in annexin V binding buffer containing 0.8 μg/ml annexin V and 0.5 μg/ml propidium iodide. After washing, cells were evaluated by flow cytometry.

### Western blot

Whole vesicle lysates generated after 2 min boiling with SDS sample buffer and cell-free supernatants were developed in SDS-PAGE and transferred to nitrocellulose. Membranes were blocked with PBS containing 5% (w/v) fat-free dry milk and then incubated at 4°C with ADAM10 or Biotin-coupled SGN-35 mAbs (1 μg/mL each) and finally stained with peroxidase-coupled goat anti-mouse IgG or streptavidin, respectively. ECL-reagent was used as substrate (GE Healthcare).

## SUPPLEMENTARY MATERIALS FIGURES


