# Supplementary material for: CD30 on extracellular vesicles from malignant Hodgkin cells supports damaging of CD30 ligand-expressing bystander cells with Brentuximab-Vedotin, in vitro
Source: Oncotarget. 2016 Apr 20;7(21):30523–35. doi: 10.18632/oncotarget.8864 (PMC5058698; doi:10.18632/oncotarget.8864)
Supplement: Supplementary file 1 [file oncotarget-07-30523-s001.pdf]

CD30 on extracellular vesicles from malignant Hodgkin cells supports damaging of CD30 ligand-expressing bystander cells with Brentuximab-Vedotin, *in vitro*

Supplementary Materials

Supplementary 1

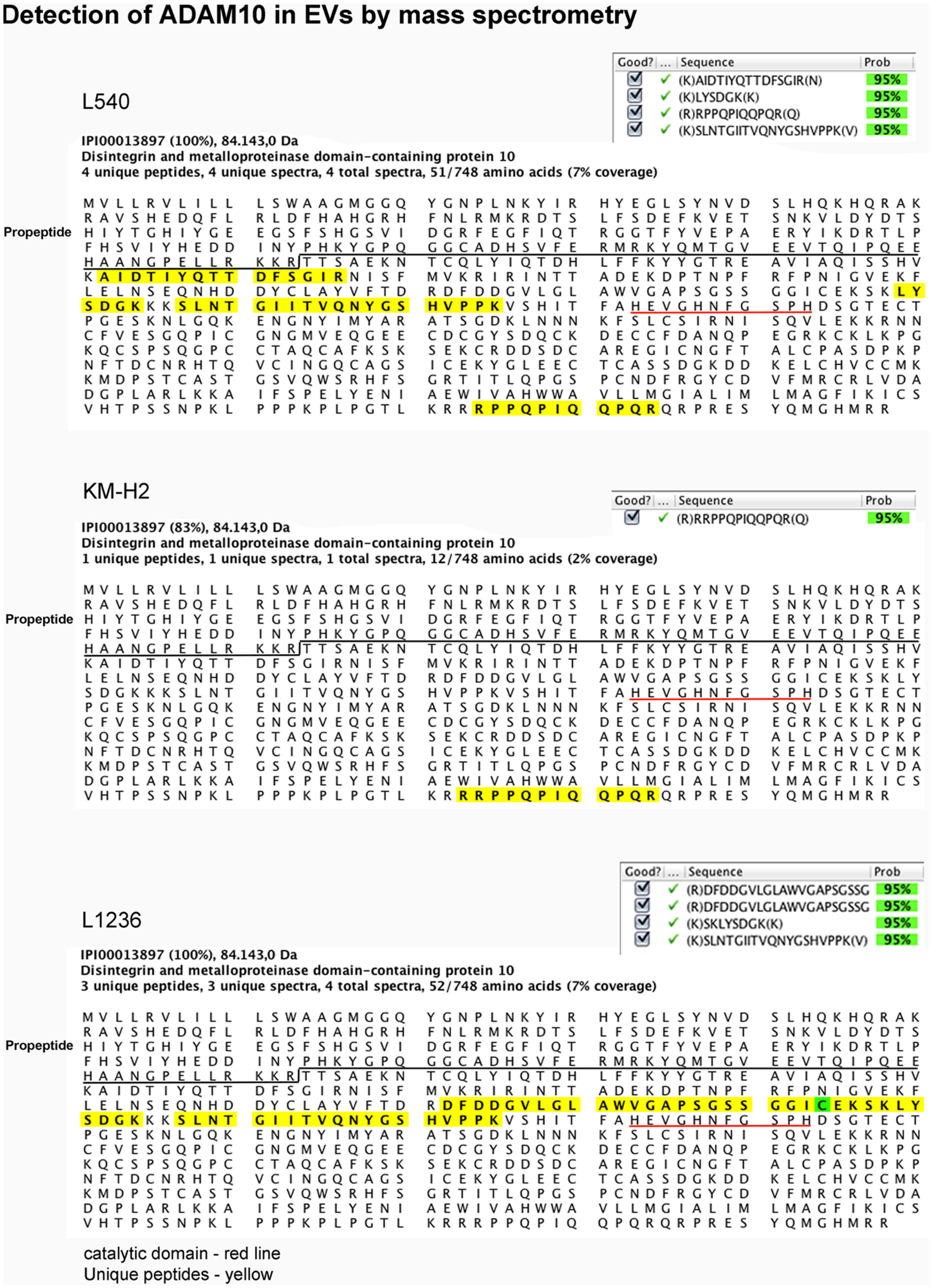

Figure: Detection of ADAM10 in cHL cell lines by mass spectrometry. Evaluation of data by Scaffold 3

Supplementary 2

Novel anti-human CD30endo monoclonal antibodies

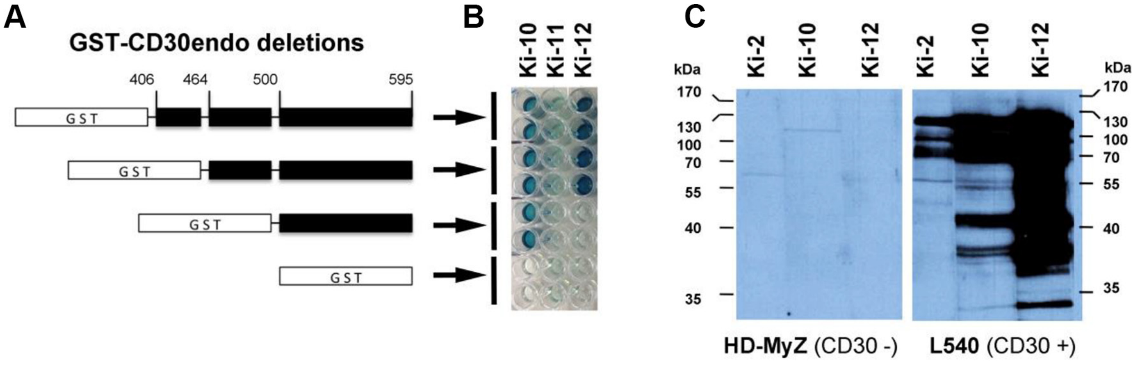

Figure: Test of anti-human CD30endo monoclonal antibodies. (**A**) GST fusion proteins were produced and used to coat microtiter plates (1 µg/mL protein in 50 mM carbonate buffer, pH 9.2). Culture supernatants of hybridomas were applied as indicated (**B**). Binding was detected by horseradish peroxidase-coupled anti-mouse IgG. (**C**) Western blot of L540 (CD30-positive) and HD-MyZ (CD30-negative; control) cell lysates with culture supernatants of the anti-human CD30endo hybridomas Ki-10 and Ki-12. The Ki-2 mAb detects the CD30 ectodomain and serves as positive control.

MATERIALS AND METHODS

Immunization and production of anti-endoCD30 monoclonal antibodies

BALB/c mice (3–4 months old, Charles River, Cologne, Germany) received primary *i.p.* immunizations with 100 µg of the purified recombinant fusion protein containing glutathion-S-transferase and the endoplasmic domain of human CD30 (GST-humCD30$^{AA406-595}$), which was dissolved in 60 µL PBS and emulsified with 40 μL of Gerbu adjuvant MM (Gerbu Biotech., Heidelberg, Germany). The mice were boosted *i.p.* on day 7, 14 and 21 with 50 µg of CD30-GST fusion protein emulsified with 20% of the adjuvant. The last two doses (50 µg of CD30-GST) were performed on day 28 and 29 without adjuvant, while fusion was done on day 30.

Spleen cells from immunized animals were collected and fused with Ag8.653 myeloma cells using polyethylene glycol 1500 (Roche Diagnostics, Mannheim, Germany). The fused cells were cultured in selection medium (HAT, Sigma-Aldrich, Deisenhofen, Germany) for 10 days. The antibodies were selected for reactivity with parts of the human CD30 endodomain, GST-humCD30$^{AA406-595}$, GST-humCD30$^{AA464-595}$, GST-humCD30$^{AA500-595}$ and with GST alone.

Binding of novel monoclonal anti-CD30 endodomain antibodies to cHL tissue

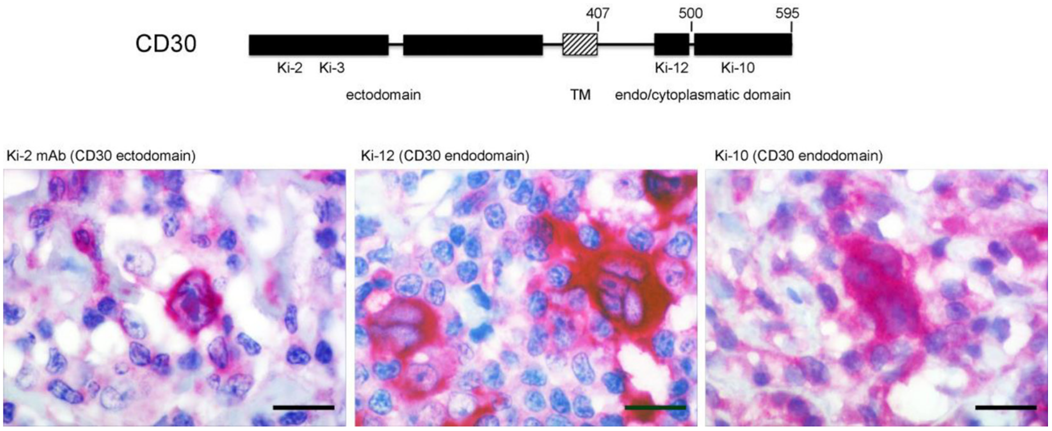

Figure: Binding sites of established and novel anti-CD30 antibodies and cHL tissue staining. Tissue sections of lymph nodes infiltrated by cHL (mixed cellularity subtype) were stained with the novel anti-CD30endo antibodies Ki-10 and Ki-12 as well as with the established anti-CD30ecto antibody Ki-2. Light microscopic image of anti-CD30 APAAP-stained thin tissue section shows CD30^{+} H-RS cell with protrusions. The bar indicates 25 µm.

Supplementary 3

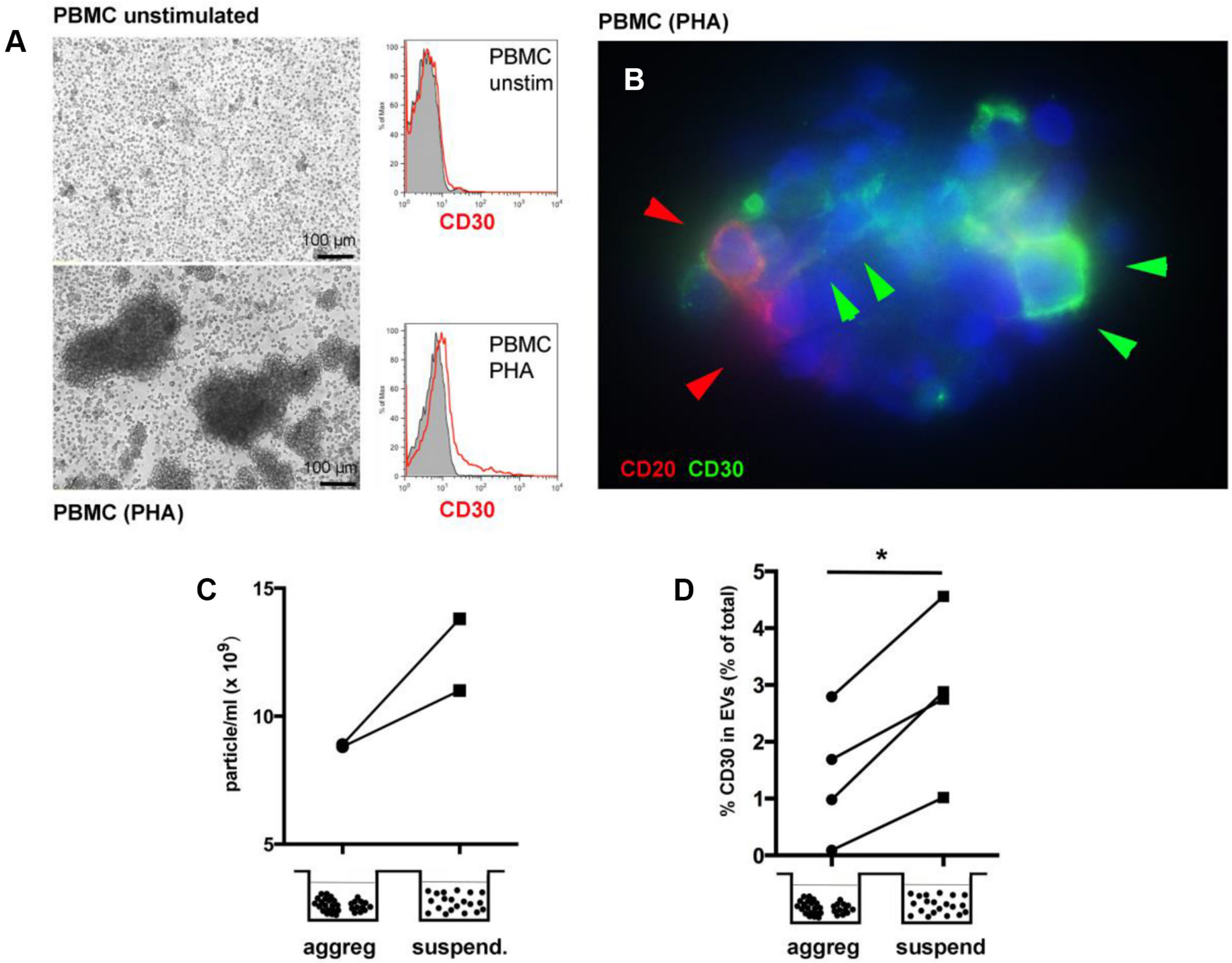

Figure: Release of extracellular vesicles from cell aggregates. (**A**) PBMCs were stimulated for 72 h with 0,1% phytohemeagglutinin (PHA). They form large aggregates upon stimulation. CD30 and CD20 expression is demonstrated by flow cytometry and immune histology (**B**). (**C**) Particles were counted by NTA in the supernatant of PBMC aggregates and suspended aggregates from two different healthy donors. (**D**) The percentage of CD30 on EVs was determined in the supernatants of aggregates and suspended aggregates from 6 different donors. The percentages of CD30EV in the supernatant of aggregates and suspended aggregates were statistically evaluated by a paired, two-tailed *t*-test ($P = 0.0103$; $N = 4$).
